# Single, 14-Day, and 13-Week Repeated Dose Toxicity Studies of Daily Oral *Gelidium elegans* Extract Administration to Rats

**DOI:** 10.3390/molecules23010217

**Published:** 2018-01-20

**Authors:** Jia Choi, Su-Jung Ryu, Kui-Jin Kim, Hyung-Min Kim, Hee-Chul Chung, Boo-Yong Lee

**Affiliations:** 1 Department of Food Science and Biotechnology, College of Life Science, CHA University, Seongnam, Kyonggi 13488, Korea; wldk3176@gmail.com (J.C.); fkadldl@naver.com (S.-J.R.); Kuijin.Kim@cha.ac.kr (K.-J.K.); 2 Newtree, Seongnam, Kyonggi, 127-16 Korea; hmkim@inewtree.com (H.-M.K.); hchung@inewtree.com (H.-C.C.)

**Keywords:** *Gelidium elegans*, *Gelidium amansii*, polyphenol-rich dietary source, repeated dose toxicity, no observed adverse effect level

## Abstract

*Gelidium elegans* extract (GEE) is derived from a red alga from the Asia–Pacific region, which has antioxidant, anti-adipogenic, and anti-hyperglycemic effects. However, detailed studies of the toxicology of GEE have not been performed. We evaluated the single oral dose toxicity of GEE in male and female Sprague-Dawley (CD) rats. GEE did not cause deaths or have toxic effects at dosages of 5000 mg/kg/day, although compound-colored stools and diarrhea were observed in both sexes, which lasted <2 days. Therefore, the LD_50_ of GEE is likely to be >5000 mg/kg. We next evaluated the repeated oral dose toxicity of GEE in CD rats over 14 days and 13 weeks. GEE did not induce any significant toxicological changes in either sex at 2000 mg/kg/day. Repeated oral dose toxicity studies showed no adverse effects, in terms of clinical signs, mortality, body mass, food consumption, ophthalmic examination, urinalysis, hematology, serum biochemistry, necropsy, organ masses, or histopathology, at dosages of 500, 1000, or 2000 mg/kg/day. The no observed adverse effect level (NOAEL) for GEE is thus likely to be >2000 mg/kg/day, and no pathology was identified in potential target organs. Therefore, this study indicates that repeated oral dosing with GEE is safe in CD rats.

## 1. Introduction

Seaweeds have become important functional components in the food industry [1]. *Gelidium elegans* (GE), also known as *Gelidium amansii*, is a seaweed that is widely distributed around the Asia–Pacific region [2]. GE contains various bioactive polyphenols and phenolic compounds, such as hesperidin [3,4]. GE has been consumed for hundreds of years and has physiological and pharmacological effects [5]. GE is also widely consumed in meals and teas or as a medicinal herb in Korean folk medicine. Given the current popularity of traditional foods, a comprehensive analysis of the associated safety issues is required.

Recently, a number of studies have demonstrated the pharmacological effects of *Gelidium elegans* extract (GEE), including antioxidant and anti-inflammatory effects, and an impact on glucose (GLU) homeostasis [6,7]. More specifically, GEE reduced the expression of key adipogenic genes, including peroxisome proliferator-activated receptor-γ (PPARγ) and CAAT/enhancer binding protein α (C/EBPα), which is necessary for the late stage of adipogenesis, and promoted expression of genes involved in energy expenditure, such as uncoupling protein 1 and PR domain containing 16 (PRDM16). All of these are important for mediating lipid turnover [2]. Moreover, our previous study showed that GEE has the potential to regulate glucose metabolism by modulating mitogen-activated protein kinase (MAPK) and phosphoinositol 3-kinase (PI3K)/protein kinase B (Akt) signaling pathways in diabetic mice [8].

We recently reported a genotoxicity study that included bacterial reverse mutation assays, chromosome aberration assays, and micronucleus assays, conducted in vitro and in vivo [9]. This showed that GEE did not induce bacterial reverse mutations, alter the frequency of micronucleated bone marrow polychromatic erythrocytes, or cause chromosomal aberrations.

However, although the genotoxicity profile of GEE has been comprehensively established, the toxicological effects of GEE following repeated administration have not been fully investigated, and thus its safety is not guaranteed. Therefore, this study aimed to evaluate the single dose, 14-day, and 13-week repeated oral dose toxicity of GEE in Sprague-Dawley (CD) rats.

## 2. Results

### 2.1. Acute Oral Toxicity Study

To determine the toxicity and the lethal dose (LD_50_) of GEE (Table 1), it was administered orally to male and female CD rats, in single doses of 0 (vehicle control) or 5000 mg/kg. During the 14-day experimental period, mortality and body mass were monitored, and necropsy was undertaken at the end of the experimental period. No deaths or clinical signs were observed. In addition, there were no significant differences in the body mass of the GEE-treated groups compared with the vehicle control group. No abnormalities were found during necropsy, but compound-colored stools and diarrhea were observed in both males and females that were given 5000 mg/kg GEE. However, these animals recovered within 2 days. Therefore, the LD_50_ of GEE is estimated to be >5000 mg/kg.

### 2.2. 14 Day Repeated Oral Dose Toxicity Study

**Figure 1:**
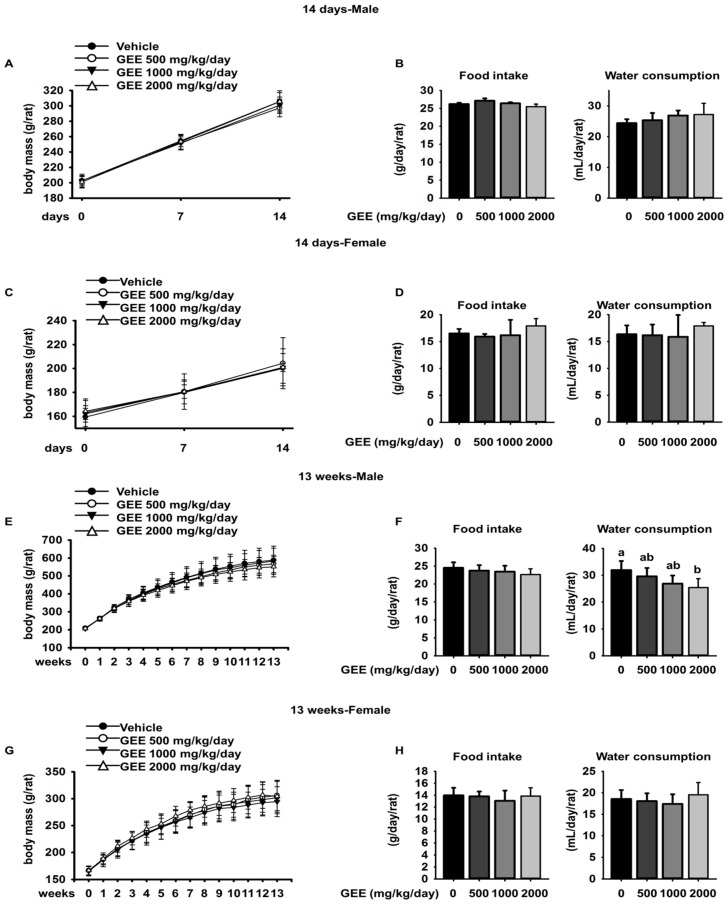
Effect of *Gelidium elegans* extract (GEE) administration on the body mass, food intake, and water consumption of rats at dosages of 500, 1000, or 2000 mg/kg/day GEE for 14 days (**A**–**D**) and for 13 weeks (**E**–**H**). Data are presented as mean ± SD (*n* = 10, ^a^ *p* < 0.05, ^b^ *p* < 0.01).

To determine the appropriate dose of GEE for the 13-week repeated oral dose toxicity study, male and female CD rats were administered GEE orally for 14 days at dosages of 0 (vehicle control), 500, 1000, or 2000 mg/kg/day. During the experimental period, mortality, clinical signs, body mass, food intake, water consumption, ophthalmic examination, hematology, serum biochemistry, urine biochemistry, and organ masses were evaluated. During the treatment period, no deaths or clinical signs related to the administration of GEE were observed. In addition, there were no significant differences in body mass, food intake, or water consumption between the GEE-treated groups and the vehicle-treated group (Figure 1A–D). Ophthalmic examination showed no abnormalities in any of the rats.

GEE had no significant hematologic or serum biochemical effects (Table 2 and Table 3). Absolute organ mass was similarly unaffected in both males and females of all test groups (Table 4). Urinalysis also revealed no significant differences in either male or female rats (Table 5). Based on the results of this study, a dosage of ≤2000 mg/kg/day for the 13-week repeated administration trial was appropriate.

### 2.3. 13 Week Repeated Oral Dose Toxicity Study

#### 2.3.1. Mortality, Clinical Signs, Body Mass, Food Intake, and Water Consumption

No deaths were caused by any of the doses of GEE tested. Gross external examination revealed no abnormalities during the experimental period, and hair quality and quantity were not affected by the treatments. Compound-colored feces were observed in six rats of both sexes in the 2000 mg/kg/day group on day 5, and in all rats of both sexes in this group from day 6 to the end of the treatment period. This was also observed in both sexes from day 12 of administration of 1000 mg/kg/day GEE. However, no other clinical signs were observed. There were no significant differences in body mass between the GEE-treated groups and the vehicle-treated group (Figure 1E,G). There were no significant differences in food intake between the groups administered GEE and the vehicle group, in either sex. In addition, there were no significant differences in water consumption in female rats, but lower water consumption was observed in males given 2000 mg/kg/day GEE throughout the treatment period, compared to controls (Figure 1F,H).

#### 2.3.2. Ophthalmic Examination, Hematology, and Serum Biochemistry

No ophthalmic disorders were found in any of the rats. GEE did not increase the plasma activity of serum aminotransferase enzymes (alanine aminotransferase (ALT) or aspartate aminotransferase (AST)), implying a lack of acute liver damage, but the average hematocrit (HCT) of females in the 1000 mg/kg/day group was significantly lower than that of the vehicle group. The percentage of basophils (BASO (%)) in females in the 0.5 g/kg/day group was significantly lower than that of the vehicle group (Table 6).

However, GEE administration did not affect red blood cell (RBC) count, hemoglobin (HGB), hematocrit (HCT), mean corpuscular volume (MCV), mean corpuscular hemoglobin (MCH), mean corpuscular hemoglobin concentration (MCHC), platelet count (PLT), white blood cell (WBC) count, neutrophils (NEU), lymphocytes (LYM), monocytes (MONO), large unstained cells (LUC), or eosinophils (EOS). In serum biochemistry, [Cl^−^] in males given 2000 mg/kg/day was slightly higher than that of the vehicle group, and [Na^+^] of the females being given 2000 mg/kg/day was also slightly higher than that of the vehicle group (Table 7). However, the differences were very small and both mean values were within the normal range. GEE treatment had no effect on other serum biochemistry parameters.

#### 2.3.3. Organ Masses, Histopathology, and Urinalysis

There were no significant effects of treatment on absolute organ mass in either males or females in all test groups (Table 8).

No pathological signs were observed (data not shown). GEE administration did not affect the histological appearance of the liver or its lining, nor did the treatments affect other vital organs (brain, lung, heart, spleen kidney, or liver). Similarly, GEE administration did not affect urinary parameters (Table 9).

## 3. Discussion

GEE is an ingredient that is often ingested daily in the diet, in much larger amounts than would be the case with a food additive [1]. It has been shown to have anti-inflammatory, antioxidant, anti-obesity, and anti-hyperglycemic effects in rodent studies [6,10,11]. However, these beneficial effects, as well as the safety of GEE, require verification in human clinical trials. Prior to such trials, it is necessary to determine the safety of GEE in animal models, using toxicity studies [12].

To determine the appropriate dose of GEE for a 13-week repeated oral dose toxicity trial, we conducted an acute oral toxicity study and a 14-day repeated dose oral toxicity study in rats.

A single oral dose of 5000 mg/kg GEE did not cause any mortalities in the rats. Thus, the oral median lethal dose (LD_50_) value for GEE is >5000 mg/kg for rats. The choice of dosages for the 14-day toxicity study (500, 1000, and 2000 mg/kg/day) were based on previous pharmacological testing, during which the efficacy of GEE was demonstrated using oral administration of 200 mg/kg/day to high fat diet fed mice [2].

GEE was administered by gavage to four groups of CD rats, each group consisting of five males and five females, once daily, for 14 days, at dosages of 0 (vehicle), 500, 1000, or 2000 mg/kg/day. There were no deaths during the administration period in any of the study groups. No GEE-induced effects were observed, in terms of clinical signs, body mass changes, changes in food or water consumption, ophthalmology, or necropsy. Thus, when GEE was orally administered repeatedly for 14 days, no systemic toxicological changes were observed. Therefore, we performed a 13-week repeated dose toxicity study using 2000 mg/kg/day GEE.

GEE was administered by gavage to four groups of CD rats (each consisting of ten males and ten females), once daily, for 13 weeks, at dosages of 0 (vehicle), 500, 1000 or 2000 mg/kg/day. This study complied with the test guidelines in the national toxicology program (NTP) protocol. There were no deaths during the administration period in any of the study groups, and GEE was well tolerated, with the treated rats showing no differences from controls in their body mass, food intake, organ masses, serum biochemistry, ophthalmic examination, urinalysis, or necropsy, and no clinical signs or abnormal behavior were noted.

Compound-colored feces were observed in both sexes at a GEE dosage of 2000 mg/kg/day, which was presumed to result from coloring by the test substance or its metabolites during excretion, because this was observed only during the treatment period. However, this finding was not toxicologically noteworthy because no treatment-induced effects on body mass or the gastrointestinal tract were observed (data not shown) [13]. The significantly lower water consumption observed in the male rats on GEE 2000 mg/kg/day was considered to be a treatment-related effect because it was observed continuously throughout the treatment period [14]. Our previous study also showed lower water consumption in the test groups, probably caused by relatively poor palatability of the GEE extract. A voluntary reduction in water intake normally causes an increase in urea nitrogen concentration [15]. Many studies also report that water consumption is correlated with urinary [Na^+^] and [Cl^−^] [16,17]. However, our data suggest that the lower water consumption and related findings were not toxicologically significant because they were within the limits of normal biological variation and were not associated with systemic abnormalities, such as abnormal serum biochemistry or changes in absolute organ mass. In addition, the difference in water consumption observed between groups of female rats was not considered to be treatment-related because it was not dose-dependent.

Female rats administered with 500 mg/kg/day GEE had a lower percentage of basophils, which are circulating blood granulocytes that are involved in inflammatory processes and comprise ~0.1–0.3% of WBCs [18,19]. However, the lower BASO (%) and related findings were not considered to be toxicologically significant because they were within the normal range for rats [20] and were unaccompanied by differences in the WBC count [21]. In addition, higher NEU and LYM counts were observed in females treated with 500 mg/kg/day, but these were also not considered to be toxicologically significant because they were within the normal range [22]. With regard to blood biochemistry, AST, ALT, and alkaline phosphatase (ALP) activities did not differ between groups, suggesting an absence of acute liver pathology [23]. There were no differences in the ratio between the vehicle-treated group and rats administered with the lower doses of GEE, and no histopathological findings, and therefore these differences were judged to be unrelated to GEE administration.

Histopathology of the uterus revealed luminal dilatation in two animals in the vehicle group and in three, four, and three animals that had been administered 500, 1000, and 2000 mg/kg/day GEE, respectively; thus, there was no difference between groups. Indeed, luminal dilatation has occasionally been reported as an incidental histopathological finding [24,25].

Romaris et al. previously demonstrated a high bioavailability of iron in a red alga seaweed in an in vitro study, which also had high dialyzable bromine percentages [26]. Moreover, Bouhlal Rhimou et al. investigated the UV protective effect of *Gelidium amansii* and showed that a *Gelidium amansii* extract, enriched by a biosorption process, increased absorption over the control group [27]. We believe that the bioavailability of GEE improves the strength of the scientific evidence provided by our study.

Based on body surface area, a dose of 2000 mg/kg in a rat would be equivalent to ~4000 mg/kg in a mouse [28]. The results of this and previous studies show that repeated oral administration of GEE does not cause any toxic effects in rats at a dosage 10-fold higher than the dosage of GEE (200 mg/kg/day) that has anti-obesity effects when administered for 7 weeks [2]. The dosage of 200 mg/kg/day GEE, which we used for mice in our previous study, is equivalent to ~1 g/60 kg/day in human adults [28]. Based on these findings, it can be surmised that the expected effective dose of GEE should involve a sufficient safety margin in humans. Taken together, these results of single, 14 days, and 13 weeks repeated oral toxicity studies confirmed that GEE was devoid of toxicological effects under our experimental conditions. Therefore, we suggested that GEE could be considered a safe dietary ingredient.

## 4. Materials and Methods

### 4.1. Preparation of Gelidium elegans Extract (GEE)

**Figure 2:**
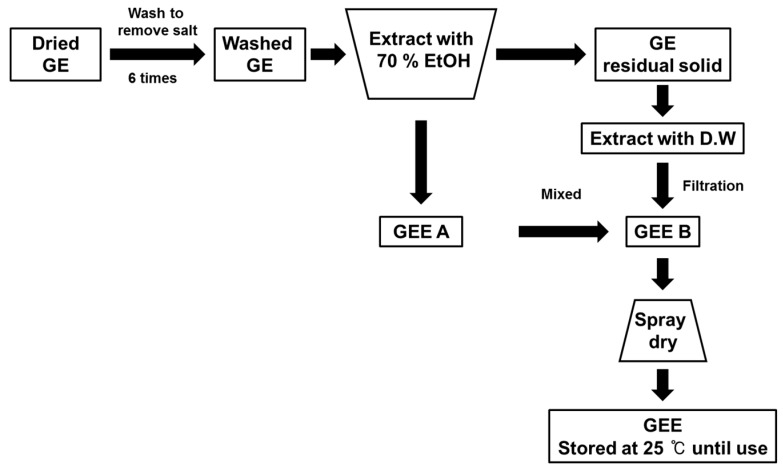
Preparation procedure for GEE [10].

To obtain the GEE, GE was washed six times, to remove sea salt, and then extracted with 70% ethanol. After these processes, the residue was extracted with deionized water at 90 °C. The ethanol and deionized water extracts were then mixed together and passed through a 50 μm filter. Following this, the spray drying method was used to prepare the final extract. The GEE was then stored at 4 °C until use (Figure 2).

### 4.2. Test Substance

GEE was provided by Newtree Co., Ltd. (Seongnam, Korea). The composition analysis showed that 1 g GEE contains 5.1% moisture, 24.1% crude ash, 16.9% crude protein, 47.6% carbohydrate, and 8.79 mg polyphenols (Table 10) [10].

### 4.3. Experimental Animals

Male and female CD rats (5 weeks old) were purchased from Orient Bio Co. (Gapyeong, Kyeonggi, Korea) and allowed free access to tap water and an irradiation-sterilized pelleted lab animal diet (Teklad Certified Irradiated Global 18% Protein Rodent Diet, 2918C, ENVIGO, Huntingdon, UK), purchased from Dooyeol Biotech (Seoul, Korea). The animals were housed individually and maintained in specific pathogen-free conditions in suspended, polycarbonate cages at 23 ± 3 °C, with a relative humidity of 55% ± 15%, a 12 h light/12 h dark cycle (08:00–20:00), 150–300 Lux luminous intensity, and 10–20 air changes per hour, during both the 14-day and 13-week repeated dose toxicity studies. After a 1-week adaptation period, the animals were randomly divided into groups. The project was approved by the Institutional Animal Care and Use Committee (IACUC Approval Number 16-R061).

### 4.4. Study Design Overview

This study was conducted in accordance with the Good Laboratory Practice (GLP) standards of the Korean MFDS (Notification No. 2014-67, MFDS, 12 February 2014), the Test Guidelines of the OECD [29], and the Korea Food and Drug Administration (KFDA) at the GLP institute, approved by the KFDA [30].

#### 4.4.1. Acute Oral Dose Toxicity Study

The acute oral toxicity of GEE was evaluated in rats, according to the procedures outlined by the OECD. Prior to dosing, rats (male and female) were fasted overnight. A single high dose of 5000 mg/kg GEE was administered orally to rats (three males and three females) in the treatment groups, while other rats (three males and three females) were given distilled water (the control group). After administration, regular observation for signs of toxicity was performed during the first 6 h after treatment and daily for a further 14 days post-administration. On day 14 after administration, the rats were euthanized.

#### 4.4.2. 14 Day Repeated Oral Dose Toxicity Study

Four groups of 40 rats (20 males and 20 females) were used. Five healthy animals of each gender received GEE once daily by oral gavage at dosages of 500, 1000, or 2000 mg/kg/day in a volume of 20 mL/kg for 14 days. Vehicle groups, treated with sterile distilled water for injection only, served as negative controls. During the period of treatment, the animals were weighed and observed daily to detect any signs of toxicity.

#### 4.4.3. 13 Week Repeated Oral Dose Toxicity Study

Four groups of 80 rats (40 males and 40 females) were used. Ten healthy animals of each gender received GEE once daily by oral gavage at dosages of 500, 1000, or 2000 mg/kg/day in a volume of 20 mL/kg for 13 weeks. Vehicle groups, treated with sterile distilled water for injection only, served as negative controls. During the period of treatment, the animals were weighed and observed daily to detect any signs of toxicity.

### 4.5. Clinical Signs

The rats were observed continuously for any clinical signs or mortality for the first hour after administration and they were checked every hour for the subsequent 6 hr. The animals were observed for clinical signs, including changes in appearance, posture, movement, urine, body surface, and fluid secretion/excretion, throughout the experimental period.

### 4.6. Body Mass

Animals were weighed on the first day (before administration, day 1) of administration and once weekly thereafter.

### 4.7. Food and Water Consumption

Food and water consumption were measured on the initial day of treatment, and then once weekly during the experimental period. The amounts of food and water placed in each cage were measured, as were the amounts remaining the next day, to calculate the daily food intake and water consumption (g/rat/day).

### 4.8. Ophthalmic Examination

During the final week of the experiment, the external parts of the eyes were examined in five males and five females per group, and then a mydriatic (Alcon Korea, Seoul, Korea) was administered to examine the rostral parts of the eye, optic media, and ocular fundus, using an ophthalmoscope and a fundus camera (Keeler Instruments Inc., Broomall, PA, USA). No abnormal signs were observed during the ophthalmic examination; therefore, no further ocular examinations were conducted.

### 4.9. Hematology and Serum Biochemistry

Blood samples were collected from the caudal vena cava of all animals scheduled for necropsy under deep isoflurane anesthesia (Kyongbo Pharm. Co., Ltd., Seoul, Korea). The animals were fasted overnight (with water available) and anesthetized with isoflurane, before blood was collected into tubes, containing potassium EDTA as an anticoagulant, for hematologic analysis, using a total hematology system (ADVIA 2120 System, Siemens Healthcare Diagnostics K.K., Munich, Germany) (red blood cell (RBC) count, hemoglobin (HGB), hematocrit (HCT), mean corpuscular volume (MCV), mean corpuscular hemoglobin (MCH), mean corpuscular hemoglobin concentration (MCHC), platelet count (PLT), white blood cell (WBC) count, and white blood cell differential based on percentage, including neutrophils (NEU), lymphocytes (LYM), monocytes (MONO), large unstained cells (LUC), eosinophils (EOS), and basophils (BASO)).

Standard serum biochemistry parameters were analyzed using an automated blood coagulation analyzer (CS2000i System, Sysmex Corporation, Tokyo, Japan). Approximately 1 mL blood was collected from each animal into tubes containing heparin for biochemical analysis, which employed an automated clinical chemistry analyzer (TBA-120FR, Toshiba Medical Systems, Inc., Tokyo, Japan) (aspartate aminotransferase (AST), alanine aminotransferase (ALT), alkaline phosphatase (ALP), creatine phosphokinase (CPK), total bilirubin (T-BIL), glucose (GLU), total cholesterol (TCHO), triglycerides (TG), total protein (TP), albumin (ALB), albumin/globulin ratio (A/G), blood urea nitrogen (BUN), creatinine (CRE), inorganic phosphorus (IP), calcium ion (Ca^2+^), sodium ion (Na^+^), potassium ion (K^+^), and chloride ion (CI^−^)).

### 4.10. Necropsy and Organ Masses

Before necropsy, all surviving animals were fasted overnight (for 16–20 h) and anesthetized using isoflurane (Ifran liquid, Hana Pharm. Co., Seoul, Korea) inhalation. After anesthesia was confirmed, blood samples were taken from the caudal vena cava, and then the abdominal aorta and caudal vena cava were cut to euthanize the animals. An initial inspection was then made of the body surface, subcutis, head, and all internal organs of the abdominal and thoracic cavities. Next, the brain, liver, lung, heart, adrenal glands, thymus, prostate gland, testes, epididymis, ovaries, uterus, spleen, and kidneys were removed, and all paired organs were measured separately.

### 4.11. Urinalysis

During the last week of the experiment, five males and five females per group were housed in metabolic cages for urine collection. Urinalysis (general urine and urine sediment examination) was performed using ~1 mL urine. GLU, bilirubin, ketone body, pH, urobilinogen, and occult blood were evaluated in urine using Multistix (Siemens Healthcare Diagnostics K.K., Tokyo, Japan).

### 4.12. Statistical Analysis

Data are expressed as mean ± standard deviation and were analyzed by one-way analysis of variance. When significant differences were detected, Duncan’s multiple range test was used to compare the means. SPSS 12.0 K (SPSS Inc., Chicago, IL, USA) was used for all statistical analyses. *p* < 0.05 was considered to represent a statistically significant difference (signified in the Figures and tables by labels a and b).

## 5. Conclusions

In conclusion, during repeated 14-day and 13-week oral administration of GEE, no systemic toxicological changes were observed, Under the experimental conditions used, the no observed adverse effect level (NOAEL) of GEE was set at 2000 mg/kg/day and no target organ pathology was observed.

## Figures and Tables

**Table 1:** General appearance and behavior of the rats during the acute oral study.

Observation	0 (mg/kg)		5000 (mg/kg)	
	6 h (M/F)	14 Days (M/F)	6 h (M/F)	14 Days (M/F)
Skin and fur	Normal	Normal	Normal	Normal
Eyes	Normal	Normal	Normal	Normal
Behavioral patterns	Normal	Normal	Normal	Normal
Compound-colored stools	Normal	Normal	3/3	Normal
Sleep	Normal	Normal	Normal	Normal
Diarrhea	Normal	Normal	3/3	Normal

(M/F, Male/Female).

**Table 2:** Hematologic values for male and female rats exposed to 500, 1000, or 2000 mg/kg/day GEE for 14 days in a repeated oral dose toxicity study.

Hematologic Values									
**Male**					**Female**				
**Parameter**	**Dosage group (mg/kg/day)**				**Parameter**	**Dosage group (mg/kg/day)**			
	**0**	**500**	**1000**	**2000**		**0**	**500**	**1000**	**2000**
RBC (10^6^/μL)	7.12 ± 0.38	7.43 ± 0.43	7.08 ± 0.26	7.18 ± 0.32	RBC (10^6^/μL)	7.30 ± 0.50	7.11 ± 0.34	7.28 ± 0.30	7.27 ± 0.39
HGB (g/dL)	14.0 ± 0.7	14.5 ± 0.7	14.1 ± 0.5	14.3 ± 0.5	HGB (g/dL)	14.4 ± 0.3	13.9 ± 0.3	14.5 ± 0.6	14.1 ± 0.7
HCT (%)	43.3 ± 2.1	45.2 ± 2.0	43.4 ± 1.2	43.9 ± 1.4	HCT (%)	43.4 ± 1.3	41.8 ± 1.2	43.6 ± 1.5	42.1 ± 2.0
MCV (fL)	60.8 ± 0.9	60.9 ± 1.6	61.4 ± 2.2	61.2 ± 1.1	MCV (fL)	59.6 ± 2.6	58.8 ± 1.6	59.9 ± 0.6	58.1 ± 2.2
MCH (pg)	19.6 ± 0.3	19.6 ± 0.5	19.9 ± 0.7	19.8 ± 0.5	MCH (pg)	19.8 ± 1.1	19.5 ± 0.8	19.9 ± 0.5	19.4 ± 0.8
MCHC (g/dL)	32.3 ± 0.3	32.2 ± 0.2	32.5 ± 0.3	32.4 ± 0.4	MCHC (g/dL)	33.1 ± 0.5	33.2 ± 0.7	33.3 ± 0.6	33.4 ± 0.2
RDW (%)	12.6 ± 0.5	12.5 ± 0.3	12.1 ± 0.4	12.2 ± 0.5	RDW (%)	11.2 ± 0.3 ^b^	11.9 ± 0.4 ^a^	11.4 ± 0.2 ^b^	11.5 ± 0.3 ^b^
HDW (g/dL)	2.39 ± 0.16	2.42 ± 0.18	2.26 ± 0.15	2.29 ± 0.02	HDW (g/dL)	2.05 ± 0.08	2.20 ± 0.18	2.14 ± 0.16	2.24 ± 0.09
PLT (10^3^/μL)	1158.4 ± 161.9	964.2 ± 80.3	1113.6 ± 123.7	1134.6 ± 113.0	PLT (10^3^/μL)	1149.8 ± 118.0	1136.0 ± 72.1	1168.2 ± 127.4	1134.8 ± 141.8
MPV (fL)	5.14 ± 0.15	5.18 ± 0.08	5.14 ± 0.21	5.06 ± 0.11	MPV (fL)	5.00 ± 0.10	4.98 ± 0.22	4.96 ± 0.09	5.02 ± 0.18
WBC (10^3^/μL)	9.93 ± 3.38	10.98 ± 2.18	11.59 ± 2.55	9.93 ± 2.57	WBC (10^3^/μL)	7.35 ± 1.66	8.39 ± 1.42	11.00 ± 2.03	10.44 ± 3.94
NEU (10^3^/μL)	1.4 ± 0.6	1.3 ± 0.4	1.4 ± 0.4	0.8 ± 0.4	NEU (10^3^/μL)	0.7 ± 0.2	0.8 ± 0.3	1.1 ± 0.5	1.1 ± 0.5
LYM (10^3^/μL)	8.1 ± 2.6	9.1 ± 1.9	9.7 ± 2.3	8.7 ± 2.0	LYM (10^3^/μL)	6.3 ± 1.5	7.2 ± 1.3	9.4 ± 1.5	8.9 ± 3.3
MONO (10^3^/μL)	0.34 ± 0.17	0.36 ± 0.12	0.38 ± 0.14	0.30 ± 0.11	MONO (10^3^/μL)	0.19 ± 0.08	0.19 ± 0.07	0.33 ± 0.16	0.27 ± 0.14
LUC (10^3^/μL)	0.08 ± 0.04	0.10 ± 0.04	0.09 ± 0.02	0.08 ± 0.04	LUC (10^3^/μL)	0.06 ± 0.03	0.08 ± 0.02	0.12 ± 0.03	0.11 ± 0.07
EOS (10^3^/μL)	0.06 ± 0.03	0.06 ± 0.01	0.06 ± 0.02	0.04 ± 0.04	EOS (10^3^/μL)	0.08 ± 0.03	0.07 ± 0.02	0.08 ± 0.04	0.08 ± 0.05
BASO (10^3^/μL)	0.02 ± 0.02	0.03 ± 0.01	0.03 ± 0.01	0.02 ± 0.01	BASO (10^3^/μL)	0.01 ± 0.01	0.02 ± 0.01	0.03 ± 0.01	0.03 ± 0.02

Data are presented as mean ± SD (*n* = 10, ^a^ *p* < 0.05, ^b^ *p* < 0.01).

**Table 3:** Serum biochemical parameters in male and female rats given 500, 1000, or 2000 mg/kg/day GEE for 14 days in a repeated oral dose toxicity study.

Serum Biochemical Parameters									
**Male**					**Female**				
**Parameter**	**DOSAGE GROUP (mg/kg/day)**				**Parameter**	**DOSAGE GROUP (mg/kg/day)**			
	**0**	**500**	**1000**	**2000**		**0**	**500**	**1000**	**2000**
AST (U/L)	83.0 ± 8.8	100.4 ± 11.0*	95.3 ± 9.1	91.2 ± 10.8	AST (U/L)	77.9 ± 6.6	88.2 ± 16.3	88.5 ± 6.2	79.7 ± 13.9
ALT (U/L)	25.8 ± 2.8 ^b^	31.2 ± 1.9 ^a^	32.7 ± 4.9^a^	27.7 ± 4.0 ^b^	ALT (U/L)	20.5 ± 3.3	22.7 ± 3.4	24.4 ± 7.6	18.5 ± 3.8
ALP (U/L)	251.1 ± 28.1	308.1 ± 72.7	247.9 ± 67.3	249.2 ± 66.3	ALP (U/L)	174.1 ± 30.2	153.0 ± 57.1	165.0 ± 40.5	149.6 ± 33.8
CPK (U/L)	199.2 ± 61.0	224.4 ± 57.1	220.2 ± 73.9	227.0 ± 112.7	CPK (U/L)	156.2 ± 62.9	191.8 ± 109.6	204.4 ± 57.8	156.2 ± 73.1
TBIL (mg/dL)	0.13 ± 0.01	0.13 ± 0.01	0.15 ± 0.01	0.15 ± 0.01	TBIL (mg/dL)	0.14 ± 0.01	0.14 ± 0.02	0.15 ± 0.02	0.12 ± 0.02
GLU (mg/dL)	128.2 ± 27.3	116.4 ± 10.4	117.3 ± 8.8	114.0 ± 7.7	GLU (mg/dL)	118.5 ± 7.1	119.1 ± 20.6	116.0 ± 5.5	124.8 ± 16.1
TCHO (mg/dL)	49.0 ± 5.1	61.0 ± 11.7	57.6 ± 15.8	67.8 ± 14.1	TCHO (mg/dL)	61.4 ± 14.1	65.6 ± 7.7	69.4 ± 8.9	64.4 ± 5.5
TG (mg/dL)	46.4 ± 19.4	40.6 ± 11.0	46.2 ± 15.4	48.2 ± 8.0	TG (mg/dL)	18.2 ± 2.9	24.4 ± 3.4	22.6 ± 2.9	23.6 ± 6.2
TP (g/dL)	5.43 ± 0.34	5.50 ± 0.18	5.35 ± 0.21	5.59 ± 0.17	TP (g/dL)	5.73 ± 0.21	5.42 ± 0.24	5.92 ± 0.29	5.67 ± 0.29
ALB (g/dL)	3.06 ± 0.17	3.11 ± 0.11	3.00 ± 0.10	3.09 ± 0.07	ALB (g/dL)	3.32 ± 0.14 ^a^	3.06 ± 0.12 ^b^	3.38 ± 0.24 ^a^	3.18 ± 0.16 ^a^
A/G ratio	1.30 ± 0.04	1.30 ± 0.04	1.28 ± 0.03	1.24 ± 0.08	A/G ratio	1.38 ± 0.06	1.30 ± 0.05	1.33 ± 0.07	1.28 ± 0.06
BUN (mg/dL)	12.5 ± 2.0	13.4 ± 1.8	11.7 ± 2.0	11.6 ± 1.9	BUN (mg/dL)	16.6 ± 3.8	16.7 ± 7.8	16.4 ± 4.3	15.7 ± 2.8
CRE (mg/dL)	0.40 ± 0.02	0.42 ± 0.02	0.39 ± 0.03	0.39 ± 0.01	CRE (mg/dL)	0.43 ± 0.04	0.45 ± 0.11	0.42 ± 0.04	0.43 ± 0.05
IP (mg/dL)	8.85 ± 0.54	8.62 ± 0.31	8.66 ± 0.51	8.95 ± 0.37	IP (mg/dL)	7.98 ± 0.39	8.07 ± 1.20	8.28 ± 0.32	8.17 ± 0.41
Ca^2+^ (mg/dL)	10.18 ± 0.40	10.10 ± 0.18	10.19 ± 0.30	10.29 ± 0.17	Ca^2+^ (mg/dL)	10.28 ± 0.13 ^a^	9.98 ± 0.25 ^b^	10.35 ± 0.17 ^a^	10.04 ± 0.06 ^a^
Na^+^ (mmol/L)	142.0 ± 2.3	143.7 ± 1.0	143.3 ± 1.3	142.6 ± 1.5	Na^+^ (mmol/L)	142.0 ± 1.5	141.2 ± 0.9	141.6 ± 0.8	140.2 ± 2.3
K^+^ (mmol/L)	4.55 ± 0.28	4.61 ± 0.25	4.61 ± 0.27	4.90 ± 0.22	K^+^ (mmol/L)	4.65 ± 0.18	4.55 ± 0.40	4.77 ± 0.18	4.80 ± 0.37
Cl^−^ (mmol/L)	100.6 ± 1.5 ^b^	103.1 ± 1.4 ^a^	102.0 ± 0.8 ^b^	101.1 ± 1.3 ^b^	Cl^−^ (mmol/L)	102.9 ± 0.7	102.9 ± 2.2	102.0 ± 1.4	101.3 ± 0.9

Data are presented as mean ± SD (*n* = 10, ^a^ *p* < 0.05, ^b^ *p* < 0.01).

**Table 4:** Organ masses (g) of male and female rats given 500, 1000, or 2000 mg/kg/day GEE for 14 days in a repeated oral dose toxicity study.

Organ Mass									
**Male**					**Female**				
**Parameter**	**DOSAGE GROUP (mg/kg/day)**				**Parameter**	**DOSAGE GROUP (mg/kg/day)**			
	**0**	**500**	**1000**	**2000**		**0**	**500**	**1000**	**2000**
Brain	1.9908 ± 0.127	1.8411 ± 0.0583	1.8600 ± 0.0930	1.9268 ± 0.1207	Brain	1.7817 ± 0.0491	1.8381 ± 0.0844	1.8083 ± 0.0916	1.8393 ± 0.0733
Liver	8.6867 ± 0.6709	8.3086 ± 0.5847	8.4274 ± 0.4454	8.7122 ± 0.3498	Liver	5.6089 ± 0.6423	6.2158 ± 0.9499	6.0697 ± 0.4692	6.2916 ± 0.5655
Lung	1.2767 ± 0.0698	1.2879 ± 0.0717	1.2442 ± 0.0677	1.2160 ± 0.0922	Lung	1.0667 ± 0.1454	1.0345 ± 0.1511	0.9943 ± 0.0668	0.9776 ± 0.0781
Heart	1.0914 ± 0.0980	1.0828 ± 0.0992	1.0728 ± 0.1155	1.0801 ± 0.0846	Heart	0.7640 ± 0.0313	0.7465 ± 0.0492	0.7684 ± 0.0234	0.7604 ± 0.0418
Adrenal gland (left)	0.0296 ± 0.0040	0.0269 ± 0.0037	0.0272 ± 0.0013	0.0286 ± 0.0032	Adrenal gland (left)	0.0307 ± 0.0009	0.0318 ± 0.0037	0.0367 ± 0.0053	0.0308 ± 0.0045
Adrenal gland (right)	0.0288 ± 0.0028	0.0262 ± 0.0023	0.0275 ± 0.0011	0.0259 ± 0.0041	Adrenal gland (right)	0.0286 ± 0.0027	0.0315 ± 0.0021	0.0352 ± 0.0085	0.0297 ± 0.0031
Thymus	0.5798 ± 0.1385	0.5142 ± 0.0747	0.5884 ± 0.1450	0.5601 ± 0.0620	Thymus	0.4132 ± 0.1156	0.4396 ± 0.1106	0.4600 ± 0.0761	0.4084 ± 0.0879
Prostate gland	0.2975 ± 0.0776	0.3365 ± 0.0979	0.3012 ± 0.0938	0.3969 ± 0.0651	Ovary (left)	0.0320 ± 0.0051	0.0335 ± 0.0056	0.0398 ± 0.0066	0.0401 ± 0.0078
Testis (left)	1.5784 ± 0.1467	1.4622 ± 0.1154	1.4963 ± 0.1423	1.5880 ± 0.1190	Ovary (right)	0.0354 ± 0.0041	0.0370 ± 0.0033	0.0365 ± 0.0037	0.0384 ± 0.0063
Testis (right)	1.5844 ± 0.1562	1.4693 ± 0.1179	1.5258 ± 0.1786	1.6005 ± 0.1273	Uterus and cervix	0.3542 ± 0.0425	0.4892 ± 0.1713	0.3866 ± 0.0548	0.4678 ± 0.1171
Epididymis (left)	0.3127 ± 0.0348	0.3114 ± 0.0455	0.3223 ± 0.0712	0.3036 ± 0.0359					
Epididymis (right)	0.3298 ± 0.0397	0.3287 ± 0.0497	0.3296 ± 0.0359	0.3073 ± 0.0434					
Spleen	0.6983 ± 0.1169	0.6959 ± 0.1093	0.7273 ± 0.0763	0.6948 ± 0.1020	Spleen	0.3974 ± 0.0681	0.4780 ± 0.1353	0.4525 ± 0.0152	0.4598 ± 0.0719
Kidney (left)	1.0931 ± 0.0698	1.1250 ± 0.0853	1.1321 ± 0.0920	1.1686 ± 0.0695	Kidney (left)	0.7273 ± 0.0711	0.8073 ± 0.1017	0.8012 ± 0.026	0.8245 ± 0.0389
Kidney (right)	1.1054 ± 0.0618	1.1423 ± 0.0927	1.1642 ± 0.0705	1.1781 ± 0.0816	Kidney (right)	0.7821 ± 0.0857	0.8424 ± 0.1099	0.8441 ± 0.0500	0.8502 ± 0.0479

Data are presented as mean ± SD (*n* = 10).

**Table 5:** Urinalysis findings in male and female rats given 500, 1000, or 2000 mg/kg/day GEE for 14 days in a repeated oral dose toxicity study.

Urinalysis Findings													
**Male**							**Female**						
**Parameter**	**Result**	**Grade**	**DOSAGE GROUP (mg/kg/day)**				**Parameter**	**Result**		**DOSAGE GROUP (mg/kg/day)**			
			**0**	**500**	**1000**	**2000**				**0**	**500**	**1000**	**2000**
Glucose (mg/dL)	Negative		5	5	5	5	Glucose (mg/dL)	Negative		5	5	5	5
Bilirubin	Negative		5	5	5	5	Bilirubin	Negative		5	5	5	5
Ketone body (mg/dL)	-		5	5	5	1	Ketone body (mg/dL)	-		5	5	5	5
pH	7					1	pH	7					
	7.5	1						7.5	1				
	8	2	4	1	1	1		8	2		1		2
	8.5	3	1	4	4	3		8.5	3	5	4	5	3
	≥9.0	4						≥9.0	4				
Urobilinogen (Ehrlich unit/dL)	0.2		5	5	5	5	Urobilinogen (Ehrlich unit/dL)	0.2		5	5	5	5
Occult blood	Negative		4	5	4	5	Occult blood	Negative		4	5	5	5
	Trace	1	1		1			Trace	1	1			
	Small	2						Small	2				

**Table 6:** Hematologic values in male and female rats given 500, 1000, or 2000 mg/kg/day GEE for 13 weeks in a repeated oral dose toxicity study.

Hematologic Values									
**Male**					**Female**				
**Parameter**	**DOSAGE GROUP (mg/kg/day)**				**Parameter**	**DOSAGE GROUP (mg/kg/day)**			
	**0**	**500**	**1000**	**2000**		**0**	**500**	**1000**	**2000**
RBC (10^6^/mL)	8.40 ± 0.20	8.30 ± 0.50	8.30 ± 0.30	8.20 ± 0.20	RBC (10^6^/mL)	7.79 ± 0.34	7.68 ± 0.32	7.41 ± 0.36	7.54 ± 0.36
HGB (g/dL)	14.30 ± 0.60	14.00 ± 0.60	14.40 ± 0.50	14.10 ± 0.60	HGB (g/dL)	14.30 ± 0.60 ^a^	14.00 ± 0.50 ^a,b^	13.50 ± 0.40 ^b^	13.90 ± 0.70 ^a,b^
HCT (%)	44.30 ± 1.80	43.30 ± 2.20	44.20 ± 1.30	43.40 ± 2.00	HCT (%)	42.60 ± 2.10 ^a^	41.80 ± 1.10 ^a,b^	40.50 ± 1.20 ^b^	41.60 ± 1.60 ^a,b^
MCV (fL)	52.70 ± 1.10	52.10 ± 1.30	53.00 ± 1.30	52.40 ± 1.30	MCV (fL)	54.80 ± 1.60	54.50 ± 1.30	54.70 ± 1.50	55.20 ± 1.20
MCH (pg)	17.00 ± 0.50	16.90 ± 0.60	17.30 ± 0.60	17.00 ± 0.50	MCH (pg)	18.30 ± 0.50	18.20 ± 0.40	18.30 ± 0.70	18.50 ± 0.40
MCHC (g/dL)	32.20 ± 0.40	32.40 ± 0.40	32.60 ± 0.50	32.60 ± 0.30	MCHC (g/dL)	33.40 ± 0.20	33.40 ± 0.50	33.40 ± 0.50	33.40 ± 0.50
RDW (%)	13.50 ± 1.20	13.30 ± 0.90	13.40 ± 0.70	13.00 ± 0.70	RDW (%)	11.70 ± 0.30	11.80 ± 0.50	11.70 ± 0.60	11.70 ± 0.30
HDW (g/dL)	2.50 ± 0.30	2.50 ± 0.20	2.50 ± 0.10	2.40 ± 0.10	HDW (g/dL)	2.21 ± 0.09	2.15 ± 0.14	2.15 ± 0.14	2.17 ± 0.15
PLT (10^3^/mL)	882.40 ± 88.60	947.80 ± 116.30	922.50 ± 68.70	845.80 ± 104.10	PLT (10^3^/mL)	914.20 ± 51.30	983.80 ± 114.90	927.10 ± 94.50	959.70 ± 91.80
MPV (fL)	5.00 ± 0.10	5.30 ± 0.50	5.14 ± 0.20	5.13 ± 0.20	MPV (fL)	5.15 ± 0.17	5.13 ± 0.13	5.26 ± 0.25	5.17 ± 0.31
WBC (10^3^/μL)	9.00 ± 2.25	10.69 ± 2.62	9.84 ± 2.58	10.00 ± 2.01	WBC (10^3^/μL)	5.73 ± 2.21	6.93 ± 2.26	5.23 ± 1.20	6.36 ± 1.54
NEU (10^3^/μL)	1.7 ± 0.6	2.4 ± 0.9	2.2 ± 1.4	1.9 ± 0.6	NEU (10^3^/μL)	0.8 ± 0.4	1.8 ± 1.5	0.8 ± 0.4	0.9 ± 0.3
LYM (10^3^/mL)	6.80 ± 1.90	7.80 ± 2.10	7.20 ± 2.00	7.70 ± 1.90	LYM (10^3^/mL)	4.60 ± 1.80	4.80 ± 1.50	4.20 ± 1.40	5.30 ± 1.40
MONO (10^3^/mL)	0.33 ± 0.14	0.32 ± 0.13	0.31 ± 0.10	0.35 ± 0.14	MONO (10^3^/mL)	0.16 ± 0.07	0.23 ± 0.16	0.14 ± 0.06	0.14 ± 0.05
LUC (%)	0.50 ± 0.20	0.40 ± 0.20	0.40 ± 0.20	0.40 ± 0.40	LUC (%)	0.67 ± 0.39	0.59 ± 0.29	0.51 ± 0.27	0.42 ± 0.16
EOS (10^3^/μL)	0.09 ± 0.03	0.08 ± 0.02	0.09 ± 0.04	0.84 ± 0.30	EOS (10^3^/μL)	0.07 ± 0.02	0.08 ± 0.04	0.06 ± 0.02	0.07 ± 0.04
BASO (10^3^/μL)	0.01 ± 0.01	0.02 ± 0.01	0.02 ± 0.01	0.02 ± 0.01	BASO (10^3^/μL)	0.01 ± 0.01	0.01 ± 0.00	0.00 ± 0.01	0.00 ± 0.01

Data are presented as mean ± SD (*n* = 10, ^a^ *p* < 0.05, ^b^ *p* < 0.01).

**Table 7:** Serum biochemical parameters in male and female rats given 500, 1000, or 2000 mg/kg/day GEE for 13 weeks in a repeated oral dose toxicity study.

Serum Biochemical Parameters									
**Male**					**Female**				
**Parameter**	**DOSAGE GROUP (mg/kg/day)**				**Parameter**	**DOSAGE GROUP (mg/kg/day)**			
	**0**	**500**	**1000**	**2000**		**0**	**500**	**1000**	**2000**
AST (U/L)	114.40 ± 24.70	107.80 ± 29.80	98.50 ± 20.30	94.60 ± 15.70	AST (U/L)	105.90 ± 33.20	98.50 ± 22.40	99.10 ± 18.30	87.20 ± 20.60
ALT (U/L)	28.50 ± 2.80	27.80 ± 5.90	26.40 ± 3.80	28.30 ± 5.10	ALT (U/L)	23.90 ± 5.70	30.70 ± 27.00	22.20 ± 5.50	18.50 ± 2.60
ALP (U/L)	78.30 ± 8.00	73.60 ± 11.40	82.80 ± 26.90	79.80 ± 17.80	ALP (U/L)	48.50 ± 10.70	45.10 ± 10.80	43.80 ± 17.10	36.00 ± 12.50
CPK (U/L)	323.60 ± 194.90	377.50 ± 317.00	290.90 ± 162.30	207.60 ± 105.70	CPK (U/L)	404.70 ± 326.40	258.20 ± 140.60	269.40 ± 133.80	233.60 ± 110.70
TBIL (mg/dL)	0.10 ± 0.00	0.10 ± 0.00	0.10 ± 0.00	0.10 ± 0.00	TBIL (mg/dL)	0.18 ± 0.03	0.18 ± 0.01	0.18 ± 0.02	0.17 ± 0.02
GLU (mg/dL)	142.60 ± 18.10	141.70 ± 15.10	137.50 ± 15.90	137.30 ± 19.60	GLU (mg/dL)	125.60 ± 15.20	130.80 ± 9.90	130.00 ± 12.60	137.10 ± 13.00
TCHO (mg/dL)	60.60 ± 16.10	56.20 ± 14.70	55.40 ± 13.50	49.50 ± 9.10	TCHO (mg/dL)	82.40 ± 15.80	79.40 ± 16.10	73.20 ± 22.50	69.90 ± 14.20
TG (mg/dL)	59.60 ± 19.60	65.70 ± 24.90	69.40 ± 26.60	47.50 ± 17.20	TG (mg/dL)	46.20 ± 19.10	44.80 ± 15.60	39.30 ± 10.70	38.30 ± 7.50
TP (g/dL)	5.80 ± 0.20	5.80 ± 0.20	5.80 ± 0.20	5.80 ± 0.20	TP (g/dL)	6.60 ± 0.40	6.50 ± 0.40	6.50 ± 0.40	6.40 ± 0.30
ALB (g/dL)	2.90 ± 0.10	2.90 ± 0.10	2.90 ± 0.10	2.90 ± 0.10	ALB (g/dL)	3.40 ± 0.20	3.40 ± 0.30	3.50 ± 0.30	3.40 ± 0.20
A/G ratio	0.90 ± 0.00	0.90 ± 0.00	0.90 ± 0.00	0.90 ± 0.00	A/G ratio	1.10 ± 0.00	1.10 ± 0.00	1.10 ± 0.00	1.10 ± 0.00
BUN (mg/dL)	13.00 ± 1.50	12.60 ± 1.30	12.10 ± 1.30	11.80 ± 1.30	BUN (mg/dL)	15.90 ± 2.20	16.20 ± 3.60	15.90 ± 2.20	16.50 ± 2.20
CRE (mg/dL)	0.40 ± 0.00	0.40 ± 0.00	0.40 ± 0.00	0.40 ± 0.00	CRE (mg/dL)	0.50 ± 0.00	0.50 ± 0.00	0.50 ± 0.00	0.50 ± 0.00
IP (mg/dL)	6.30 ± 0.70	6.20 ± 0.50	6.60 ± 0.30	6.60 ± 0.60	IP (mg/dL)	5.40 ± 0.70	5.40 ± 0.70	5.30 ± 0.80	5.00 ± 0.50
Ca^2+^ (mg/dL)	9.60 ± 0.30	9.50 ± 0.30	9.60 ± 0.20	9.50 ± 0.10	Ca^2+^ (mg/dL)	9.70 ± 0.30	9.80 ± 0.20	9.80 ± 0.30	9.60 ± 0.20
Na^+^ (mmol/L)	137.3 ± 1.0	136.9 ± 1.3	137.9 ± 0.9	137.2 ± 1.0	Na^+^ (mmol/L)	139.0 ± 1.4	139.6 ± 1.0	140.3 ± 0.9	140.0 ± 1.1
K^+^ (mmol/L)	4.40 ± 0.30	4.30 ± 0.10	4.50 ± 0.20	4.30 ± 0.20	K^+^ (mmol/L)	4.10 ± 0.10	4.00 ± 0.20	4.10 ± 0.20	4.10 ± 0.20
Cl^−^ (mmol/L)	103.00 ± 0.70 ^a,b^	101.80 ± 1.10 ^b^	102.60 ± 1.80 ^a,b^	103.70 ± 1.20 ^a^	Cl^−^ (mmol/L)	105.00 ± 2.40	105.30 ± 1.30	105.00 ± 1.90	104.90 ± 1.20

Data are presented as mean ± SD (*n* = 10, ^a^ *p* < 0.05, ^b^ *p* < 0.01).

**Table 8:** Organ masses (g) of male and female rats given 500, 1000, or 2000 mg/kg/day GEE for 13 weeks in a repeated oral dose toxicity study.

Organ Mass									
**Male**					**Female**				
**Parameter**	**DOSAGE GROUP (mg/kg/day)**				**Parameter**	**DOSAGE GROUP (mg/kg/day)**			
	**0**	**500**	**1000**	**2000**		**0**	**500**	**1000**	**2000**
Brain	2.1722 ± 0.0870	2.1986 ± 0.0835	2.1897 ± 0.0659	2.2072 ± 0.0692	Brain	1.9980 ± 0.0509	2.0040 ± 0.0814	2.0165 ± 0.0561	1.9942 ± 0.1344
Liver	14.0444 ± 2.0679	14.1644 ± 1.9002	14.2981 ± 2.3778	13.6100 ± 2.2241	Liver	6.9079 ± 0.8804	7.4317 ± 1.4922	7.2338 ± 0.9856	7.3973 ± 0.8108
Lung	1.6469 ± 0.1532	1.7322 ± 0.1829	1.7312 ± 0.1256	1.6717 ± 0.1063	Lung	1.3203 ± 0.1561	1.2862 ± 0.1248	1.2307 ± 0.1022	1.3026 ± 0.1534
Heart	1.6364 ± 0.2549	1.5973 ± 0.1365	1.5962 ± 0.1673	1.5401 ± 0.1419	Heart	0.9488 ± 0.0584	0.9672 ± 0.1070	0.9387 ± 0.1000	0.9532 ± 0.0945
Adrenal gland (left)	0.0317 ± 0.0065 ^a,b^	0.0328 ± 0.0058^a^	0.0282 ± 0.0059 ^a,b^	0.0271 ± 0.0023 ^b^	Adrenal gland (left)	0.0348 ± 0.0038	0.0334 ± 0.0066	0.0351 ± 0.0054	0.0322 ± 0.0048
Adrenal gland (right)	0.0302 ± 0.0055 ^a^	0.0303 ± 0.0043^a^	0.0268 ± 0.0053 ^a,b^	0.0251 ± 0.0016 ^b^	Adrenal gland (right)	0.0331 ± 0.0033	0.0314 ± 0.0059	0.0331 ± 0.0051	0.0312 ± 0.0041
Thymus	0.2616 ± 0.0819	0.2656 ± 0.0519	0.2344 ± 0.0622	0.2207 ± 0.0438	Thymus	0.2378 ± 0.0525 ^a,b^	0.2136 ± 0.0589 ^a,b^	0.2005 ± 0.0383 ^b^	0.2509 ± 0.0527 ^a^
Prostate gland	0.6121 ± 0.1828	0.6580 ± 0.2972	0.6861 ± 0.2216	0.6156 ± 0.1483	Ovary (left)	0.0397 ± 0.0058	0.0377 ± 0.0106	0.0391 ± 0.0078	0.0433 ± 0.0091
Testis (left)	1.8282 ± 0.0968	1.8374 ± 0.2510	1.8507 ± 0.1118	1.8145 ± 0.0958	Ovary (right)	0.0401 ± 0.0050	0.0370 ± 0.0089	0.0393 ± 0.0059	0.0424 ± 0.0132
Testis (right)	1.8265 ± 0.1172	1.8273 ± 0.2536	1.8414 ± 0.1086	1.8167 ± 0.1191	Uterus and cervix	0.7733 ± 0.1765	0.8494 ± 0.2801	0.8345 ± 0.3134	0.6764 ± 0.2565
Epididymis (left)	0.6682 ± 0.0414	0.7074 ± 0.0725	0.6761 ± 0.0413	0.6726 ± 0.0379					
Epididymis (right)	0.6826 ± 0.0430	0.7055 ± 0.0762	0.6746 ± 0.0484	0.6772 ± 0.0455					
Spleen	0.9013 ± 0.1871	0.9034 ± 0.1801	0.8648 ± 0.0925	0.8385 ± 0.1602	Spleen	0.5547 ± 0.1078	0.5442 ± 0.0681	0.5112 ± 0.0958	0.5308 ± 0.0741
Kidney (left)	1.5667 ± 0.1984	1.5774 ± 0.2051	1.5679 ± 0.1791	1.6136 ± 0.1419	Kidney (left)	0.8260 ± 0.0764	0.9073 ± 0.1266	0.8913 ± 0.1462	0.8989 ± 0.0966
Kidney (right)	1.5988 ± 0.1988	1.6185 ± 0.2202	1.6256 ± 0.1887	1.6591 ± 0.1470	Kidney (right)	0.8560 ± 0.0750	0.9106 ± 0.1154	0.9148 ± 0.1228	0.9151 ± 0.0940

Data are presented as mean ± SD (*n* = 10, ^a^ *p* < 0.05, ^b^ *p* < 0.01).

**Table 9:** Urinalysis findings in male and female rats given 500, 1000, or 2000 mg/kg/day GEE for 13 weeks in a repeated oral dose toxicity study.

Urinalysis Findings													
**Male**							**Female**						
**Parameter**	**Result**	**Grade**	**DOSAGE GROUP (mg/kg/day)**				**Parameter**	**Result**	**Grade**	**DOSAGE GROUP (mg/kg/day)**			
			**0**	**500**	**1000**	**2000**				**0**	**500**	**1000**	**2000**
Glucose (mg/dL)	Negative		5	5	5	5	Glucose (mg/dL)	Negative		5	5	5	5
Bilirubin	Negative		5	5	5	4	Bilirubin	Negative		5	5	5	4
	Small	1				1		Small	1				
Ketone body (mg/dL)	-		3	2	2	1	Ketone body (mg/dL)	-		5	5	4	5
	+/−	1	1	1	1	1		+/−	1			1	
	1+	2	1	2	2	3		1+	2				
pH	7						pH	7			1		
	7.5	1						7.5	1			1	
	8	2				1		8	2	4		1	
	8.5	3	5	5	3	4		8.5	3	1	4	3	4
	≥9.0	4			2			≥9.0	4				1
Urobilinogen (Ehrlich unit/dL)	0.2		5	5	5	4	Urobilinogen (Ehrlich unit/dL)	0.2		5	5	5	5
	1	1				1		1	1				
Occult blood	Negative		3	3	2	3	Occult blood	Negative		5	5	5	5
	Trace	1	2	2	1			Trace	1				
	Small	2			2	2		Small	2				

**Table 10:** Composition of GEE [10].

Nutrient	Ingredient	Content
Proximate composition (%)	Moisture	5.1
	Crude ash	24.1
	Crude protein	16.7
	Carbohydrate	47.6
Total polyphenols	8.79 mg/g	
